# Icaritin Shows Potent Anti-Leukemia Activity on Chronic Myeloid Leukemia *In Vitro* and *In Vivo* by Regulating MAPK/ERK/JNK and JAK2/STAT3 /AKT Signalings

**DOI:** 10.1371/journal.pone.0023720

**Published:** 2011-08-22

**Authors:** Jian feng Zhu, Zi jian Li, Guang sen Zhang, Kun Meng, Wen yong Kuang, Jin Li, Xin fu Zhou, Rui juan Li, Hong ling Peng, Chong wen Dai, Jian Kai Shen, Fan jie Gong, Yun xiao Xu, Su fang Liu

**Affiliations:** 1 Division of Hematology, Institute of Molecular Hematology, The Second Xiang-Ya Hospital, Central South University, Changsha, Hunan, People's Republic of China; 2 Shenogen Biomedical Co, Ltd, Beijing, Haidian, Beijing, People's Republic of China; Emory University, United States of America

## Abstract

**Purpose:**

To explore the effects of Icaritin on chronic myeloid leukemia (CML) cells and underlying mechanisms.

**Method:**

CML cells were incubated with various concentration of Icaritin for 48 hours, the cell proliferation was analyzed by MTT and the apoptosis was assessed with Annexin V and Hoechst 33258 staining. Cell hemoglobinization was determined. Western blotting was used to evaluate the expressions of MAPK/ERK/JNK signal pathway and Jak-2/Phorpho-Stat3/Phorsph-Akt network-related protein. NOD-SCID nude mice were applied to demonstrate the anti-leukemia effect of Icaritin *in vivo*.

**Results:**

Icaritin potently inhibited proliferation of K562 cells (IC50 was 8 µM) and primary CML cells (IC50 was 13.4 µM for CML-CP and 18 µM for CML-BC), induced CML cells apoptosis and promoted the erythroid differentiation of K562 cells with time-dependent manner. Furthermore, Icaritin was able to suppress the growth of primary CD34+ leukemia cells (CML) and Imatinib-resistant cells, and to induce apoptosis. In mouse leukemia model, Icaritin could prolong lifespan of NOD-SCID nude mice inoculated with K562 cells as effective as Imatinib without suppression of bone marrow. Icaritin could up-regulate phospho-JNK or phospho-C-Jun and down-regulate phospho-ERK, phospho-P-38, Jak-2, phospho-Stat3 and phospho-Akt expression with dose- or time-dependent manner. Icaritin had no influence both on c-Abl and phospho-c-Abl protein expression and mRNA levels of Bcr/Abl.

**Conclusion:**

Icaritin from Chinese herb medicine may be a potential anti-CML agent with low adverse effect. The mechanism of anti-leukemia for Icaritin is involved in the regulation of Bcr/Abl downstream signaling. Icaritin may be useful for an alternative therapeutic choice of Imatinib-resistant forms of CML.

## Introduction

Chronic myeloid leukemia (CML) is a clonal myeloproliferative disease characterized by Philadelphia chromosome, which generates Bcr/Abl fusion gene and P210 oncoprotein to produce a constitutively active tyrosine kinase. The selective tyrosine kinase inhibitor Imatinib can block this kinase activity by occupying ATP binding site of Bcr/Abl and inhibit CML cell growth effectively [1]. Based on clinical studies, including early chronic phase (CP)[2], [3], accelerated phase (AP)[4], and myeloid blast crisis (BC)[5], Imatinib is considered as an effective agents for the first-line therapy in newly diagnosed CML-CP patient[6] and a conventional treatment option for CML.

Unfortunately, resistance to Imatinib occurs frequently during CML-AP and BC, resulting in remission intervals usually lasting only 6 to 12 months. CML-BC is fundamentally different from CML-CP in many aspects. The major features that occur during CML progression are marked changes in growth, differentiation, apoptosis, and cell adhesion [7], [8]. On the other hand, blast crisis is relatively resistant to tyrosine kinase inhibitor (TKI) therapy, even the second-generation TKI [9]. Thus, novel therapeutic approaches that target signaling pathways other than Bcr/Abl are urgently needed for treatment of CML.

**Figure 1:**
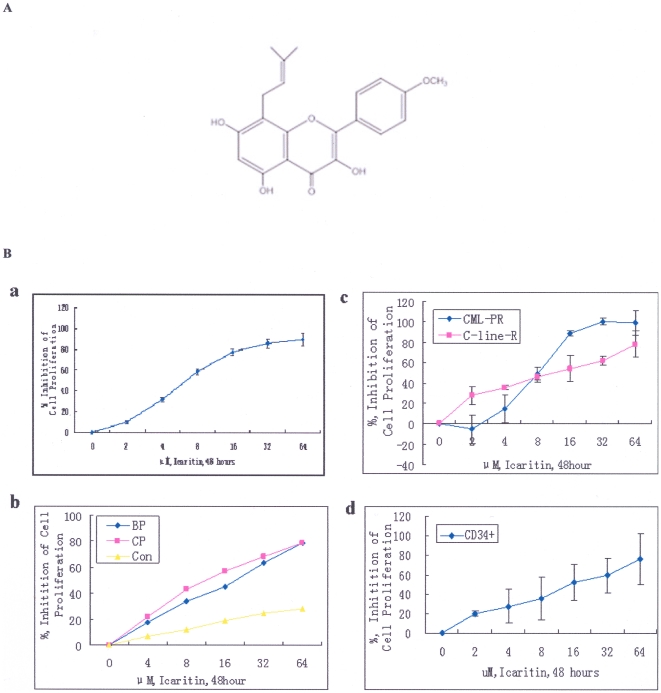
Icaritin inhibits K562 cells or primary chronic myeloid leukemic cells growth. A. Chemical structure of Icaritin. B. (a). Effects of Icaritin on K562 cell proliferation (MTT assay). (b). Effects of Icaritin on fresh primary cells (BMMCs) growth from CML patients bone marrow (CML-CP: 14; CML-BC: 6) or fresh primary cells (BMMCs) growth from normal subjects (n = 11) bone marrow (MTT assay). The values represent mean±SD of triplicate cultures. (c). Effects of Icaritin on the growth inhibition of Imatinib-resistant cells line (C-line-R) or Imatinib-resistant primary cells from one CML patient (CML-pR). (d). Proliferation-inhibition of Icaritin on CD34+ cells from 3 case of CML-BC patients.

Icaritin (Fig. 1A), a hydrolytic product of Icariin, is a constituent of the traditional Chinese herbal medicine Epimedium. Icariin can be metabolized into Icaritin by human intestinal bacteria *in vitro*, while Icaritin may exert estrogen-like activities [10]. Previous studies showed that Icariin possesses multiple kinds of biological activities, including cardiovascular function improvement, hormone regulation, modulation of immunological function and anti-tumor activity [11], [12], [13]. It has been reported that Icariin was able to induce liver cancer cell line-SMMC-7721 cells apoptosis [13], or to reverses the malignant phenotype of gastric carcinoma cell line-SGC-7901 by suppressing the adhesion and migration of cancer cells [14]. In hematological malignancy, it has been shown that Icariin not only induced differentiation of promyelocytic leukemia HL-60, but also lead to HL-60 cell apoptosis [15], which involved a down-regulated Bcl-2 and c-myc expression [16]. However, the effects of Icaritin on other types of leukemia and the underlying mechanisms remain elusive.

In this study, we investigated the effects of Icaritin on growth of the Bcr/Abl-bearing K562 [17], primary CML cells (including CD34+ cells), and imatinib-resistant cells, and found that Icaritin inhibited growth and induced apoptosis and differentiation in these cells, which provides strong rationales for potential clinical application of Icaritin as a novel agent against CML.

## Methods

### Reagents and antibodies

Icaritin with a purity of up to 99.5% was provided by Dr. Kun Meng (Shenogen Phama Group, Beijing, China). A stock solution (20 mM/L) was prepared by dissolving Icaritin in DMSO (Sigma, St. Louis, MO) and stored at −200C.Imatinib-resistant cells were obtained from Institute of zoology, Chinese Academy of Science, Beijing. Human CD34 selection kit (EasySep) was purchased from StemCell Technologies. Mitochondria isolation kit was purchased from Thermo scientific Inc. SB203580 was from Calbiochem (San Diego, CA). Antibodies for c-Abl, phospho-c-Abl, p-38, phospho-Akt(Ser473),Apaf-1(R205), Jak-2,phospho-Stat3(Tyr705), phospho-p38 were from Cell Signaling Technology (Beverly, MA). C-Jun, phospho-c-Jun, ERK, phospho-ERK, JNK, phospho-JNK, cytochrome c, caspase-3, caspae-9, Bcl-2, Bax, and β-actin antibodies were from Santa Cruz biotechnology (Santa Cruz, CA).

### Cell culture and cell proliferation assay

K562 cells were maintained in RPMI-1640 medium (Gibco) containing 10% FBS at 37°C in a humidified atmosphere of 5% CO_2_. Imatinib-resistant cells were maintained in the medium containing 1 µM Imatinib mesylate(Novartis Pharmaceuticals)and cultured in drug-free medium before all experimental procedures. Primary CML cells were harvested from bone marrow samples of CML patients and bone marrow mononuclear cells (BMMCs) were isolated by Ficoll-Paque isolation solution. The CD34-positive cells of bone marrow were isolated and purified by CD34 selection kit in 4 of patients with CML (1 case was resistant to Imatinib therapy, 3 cases were in CML-BC). CML was diagnosed according to clinical and laboratory criteria. All cases were Philadelphia chromosome positive. Total 24 CML patients were enrolled in the studies (14 CML-CP and 10 CML-BC) and 11 healthy individuals were used as controls. This study was conducted according to the principles expressed in the Declaration of Helsinki. The study was approved by the Institutional Review Board of the Second Xiangya hospital, central South University. All patients provided written informed consent for the collection of samples and subsequent analysis. Fresh bone marrow mononuclear cells were isolated by Ficoll centrifugation and re-suspended in PRMI-1640 medium supplemented with 10% FBS in an incubator.

K562, Imatinib-resistant cells at exponential growth phase, primary CML cells,primary normal bone marrow cells and primary CD34+ cells from 4 CML-BC patients were treated with Icaritin for 48 h in indicated concentrations and cell proliferation was analyzed by MTT method [18]. For time courses analysis, K562 cells were treated with 8 µM Icaritin for 2 h, 4 h, 8 h, 12 h and 24 h. IC_50_ values were determined using a nonlinear regression program calcusyn (Biosoft, Cambridge, UK). Cell viability was determined with the trypan blue dye exclusion method. Cell cycle analysis was performed as described [19].

### Apoptosis assay

K562 or Imatinib-resistant cell (5×10^4^ cell/ml) were seeded in 6-well plates and treated with different concentrations of Icaritin as indicated for 48 hours. The apoptotic morphology of K562 was evaluated by Hoechst 33258 (KeyGen Biotech Co, Ltd) following manufacturer's protocol. Cells undergoing apoptosis were assessed with an Annexin V (AV) apoptosis detection Kit I (Becton Dickinson, USA). As an evaluation of apoptosis-related cytochrome C change, Mitochondria and cytosol fraction for cultured cells were isolated by special kit following the manufacturer's instruction. Cytochrome C protein in cytosol assay (western blot) was finished.

### Cytospin, Wright-Giemsa staining, benzidine staining and flow-cytometry assay

Cells (5×10^4^ cells/ml) were spun onto a microscope slide and stained with Wright-Giemsa solution. Cell hemoglobinization was analyzed by benzidine staining as described [20]. In brief, 5×10^5^ cell/ml was mixed with 200 µl benzidine reagent dihydrochloride (Sigma). The percentage of benzidine-positive cells (blue cells) was determined by light microscopic examination. FACS Calibur (Becton Dickinson, USA) was used to determine expression of erythroid markers, such as glycophorin A (CD235a) and CD71 (Becton Dickinson, USA).

### Real-time PCR assay

Total RNA was extracted from Icaritin-treated and untreated K562 by using TRIzol reagent (Gibco, USA). Five micrograms of RNA was reverse-transcribed into cDNA using Revert Aid TM first-strand synthesis kit (Fermentas Inc, USA). Transcribed cDNA was amplified and quantified with the real-time fluorescent quantitative PCR using a Dynao SYBR Green qPCR kit (Finnzyams, Finland). β-actin and BCR/ABL primers were designed according to reference[21].

### Western blot

Cells were homogenized on ice in lysis buffer (50 mM Tris-HCL, PH 7.5, 150 mM NaCl, 1% NP-40, 0.25% Na-desoxycholate, 5 mM EDTA, 1 mM NaF, 25 mM Na3vo4, 0.1 mM PMSF and 2 µg/mL Aprotinin). After determining the concentration, equal amount of protein (∼30 µg/well) was separated on 8% or 12% SDS-PAGE and transferred onto PVDF membranes. The membranes were probed with various primary antibodies, HRP-conjugated secondary antibodies, and visualized with enhanced chemiluminescence (ECL) detection reagents (Amersham Pharmacia Biotech).

### In vivo tumor formation assays

Female NOD-SCID nude mice (6 to 8 weeks old) from Shanghai Experimental Animal Center Chinese Academy of Sciences, Shanghai, China were used according to the animal protocol. All animals were handled in strict accordance with good animal practice as defined by the relevant national and local animal welfare bodies, and all animal work was approved by the Institutional Review Board of the Second Xiangya hospital, Central South University (permit number: SYXK(xiang) 2004-0013). K562 at 2×10^6^ were injected into each mouse via tail vein. After one week, the mice inoculated with K562 were randomized into four groups (5 mice per group): (1) Untreated group as a negative control; (2) Icaritin-treated group (4 mg/kg/day); (3) Icaritin-treated group (8 mg/kg/day); (4) Imatinib-treated group (150 mg/kg/day) as a positive control. The drugs were administrated into mice by intraperitoneal injection. Peripheral blood cells were counted once a week. The percentages of leukemia cells after labeled with FITC-conjugated anti-human CD45 antibody (Becton Dickinson) were detected by flow cytometry. Survival of mice was monitored from the first day of treatment until death. The mice were euthanized when became moribund at the tenth week, all surviving mice were euthanized. Bone marrow, liver and spleen were collected, and pathological sections were prepared and stained with hematoxylin-Eosin (HE).

### Statistical analysis

The lifespan of mice was analyzed by Kaplan-Meier methods, while the leukemia cell load, WBC numbers, CD45^+^ cells and spleen weight, were analyzed with one-way ANOVA and independent sample *t* test. P values less than 0.05 were considered statistically significant and were derived from 2-tailed statistical test. All statistical treatment was performed using the software SPSS 14.0 for windows (Chicago, IL).

## Results

### 1. Icaritin inhibits proliferation of both K562, Imatinib-resistant cells and primary CML cells

To determine the effects of Icaritin on growth of CML cells, we treated cells with Icaritin at different concentrations and assessed the cell growth by MTT assay. We found that Icaritin effectively inhibited K562 proliferation (Fig. 1B-a) with rates of inhibition 31.5%, 58.2%, 77.1%, 85.6% and 89.8% for Icaritin concentration at 4, 8, 16, 32 and 64 µM, respectively. The IC_50_ value of Icaritin was 8 µM. We also tested the effects of Icaritin on growth of acute myeloid leukemia cell lines, including Raji, HL-60 and kasumi-1. The results showed the IC_50_ values for inhibiting these cells growth were 20 to 76 µM that were higher than that observed in K562. We also observed that Icaritin inhibited proliferation of primary CML cells from patients with CML-CP (14 cases) and CML-BC (6 cases). Icaritin inhibited proliferation of these CML cells in a dose-dependent manner (Fig. 1B-b). The IC_50_ values of Icaritin on these cells were 13.4 µM (CML-CP) and 18 µM (CML-BC), respectively. However, no significant effect was observed for Icaritin-treated normal bone marrow cells (Fig 1B-b).More importantly, we also found that Icaritin was able to potently inhibit the growth of both Imatinib-resistant cells strain and primary imatinib-resistant cells(CD34+) from one CML patient (Fig 1B-c), indicating Icaritin, to a certain extent, may play an role in reversing imatinib-resistance. In addition, we confirmed that Icaritin showed similar effect in proliferation-inhibition on CD34+ cells derived from CML-BC patients(Fig 1B-d).

### 2. Icaritin induces K562 apoptosis

**Figure 2:**
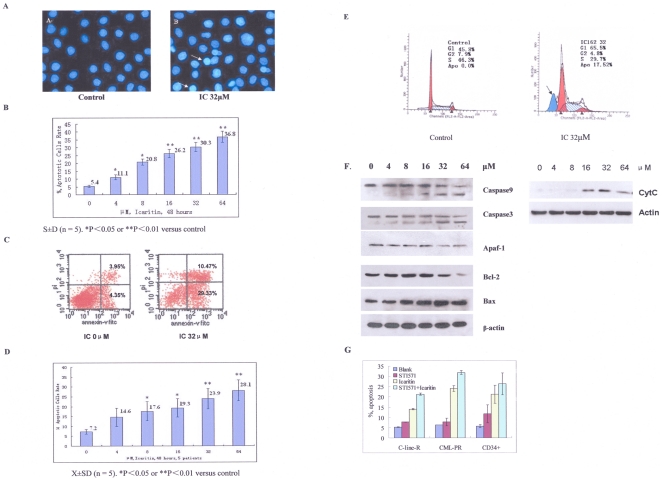
Icaritin induces K562 cells or primary cells apoptosis. A. Morphological features for apoptosis in untreated and Icaritin-treated K562 cells were revealed by Hoechst 33258 staining. Condensed chromatin and apoptotic body could be found in Icaritin-treated K562 cells by fluorescence microscope (Olympus, B×50); ×400. B. Percentages of apoptotic cells in various concentration of Icaritin-treated K562 cells (Hoechst 33258 staining). The results represent mean±SD of triplicate experiments. C. Flow-cytometry analysis showed externalized PS, revealed by Annexin staining, increased significantly in 32 µM Icaritin-treated K562 cells. D. Percentages of apoptotic cells in Icaritin-treated fresh primary cells from CML-BP patients bone marrow (n = 5) based on annexin V expression assays. Error bars represent SD of experiments. E. Cell cycle analysis showing sub-G1 content in Icaritin (32 µM)-treated cells (right panel) and control (left panel). F. Effects of Icaritin on caspase-9, caspase-3, Apaf-1, bax, bcl-2 and cyt-c protein expression (western blot results). β-actin is used as loading control. G. Effects of Icaritin on apoptosis in Imatinib-resistant cells, CD34+cells from one CML patient with Imatinib-resistance, and CD34+ cells from 3 cases with CML-BC (Annexin V analysis).

To probe the mechanisms by which Icaritin inhibited cell proliferation, we examined morphologic changes in Icaritin treated cells. K562 exposed to different concentrations of Icaritin for 48 h exhibited morphologic characteristics of apoptosis such as condensation of nuclear, as revealed by Hoechst 33258 staining (Fig. 2A) in an concentration dependent manner (Fig. 2B). Externalized PS, a characteristic of early apoptosis, as revealed with the annexin V staining, was significantly increased in Icaritin-treated K562 compared to untreated cells (Fig. 2C).

Noticeably, primary bone marrow cells from five CML-BC patients treated with Icaritin exhibited significant apoptosis in a dose-dependent manner, as revealed with the annexin V assays (Fig. 2D). Cell population in the sub-G1 phase was also increased in Icaritin-treated K562 (Fig. 2E). Western blot was performed to assess expression of Bcl-2, Bax and cytochrome C, and activation of caspase-3, caspase-9 and Apaf-1. Icaritin significantly inhibited Bcl-2 protein expression and up-regulated Bax protein expression in K562 with a dose-dependent manner accompanied by the cleavage activation of caspase-3 or caspase-9, and a down-regulated expression of Apaf-1 (Fig. 2F). To further document that the release of cytochrome C is from mitochondria, we prepared the cytosolic fraction of K562 cells, and western blot was done. The result showed that Icaritin could induce cytochrome C release with dose-dependent manner (Fig.2F). These results suggest that Icaritin induced cell apoptosis is involved in mitochondrial-mediated caspase pathway. We then examined whether Icaritin may induce CD34+ cells apoptosis in 4 cases with CML (1 for Imatinib resistance; 3 for CML-BC) and Imatinib-resistant cells line, As shown in Fig 2G, Icaritin could induce cells apoptosis significantly both on CD34+ CML and Imatinib-resistant cells.

### 3. Icaritin induces K562 to differentiate toward the erythroid lineage

**Figure 3:**
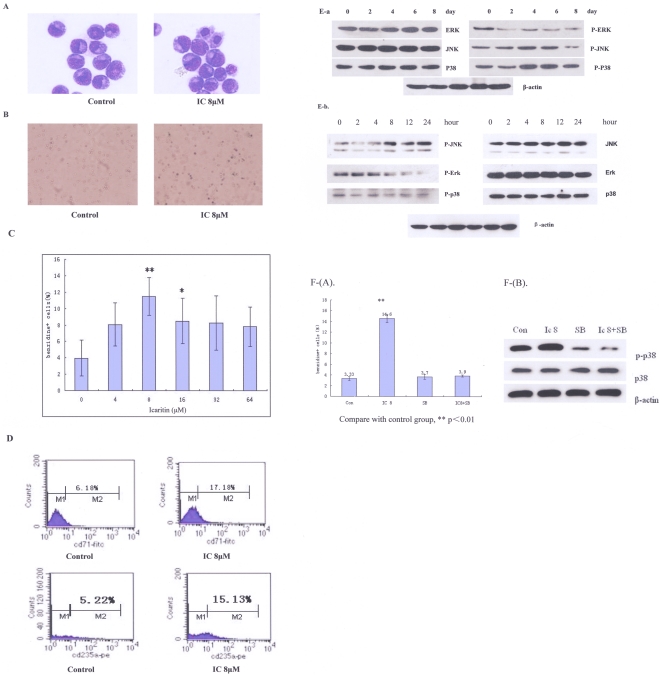
Icaritin induces K562 cells to differentiate toward the erythroid linage. A. morphologic change in K562 cells toward the erythroid differentiation.K562 cells were treated with 8 µM Icaritin, and cultured for 6 days. Then cells were stained with Wright-Giemsa and photographed (×1000). B.C. Icaritin-treated K562 cells were stained with benzidine. Compared with control (Fig B: left panel), cells exhibited deep blue appearances (Fig B: right panal) (×1000); in 8 µM Icaritin concentration, the percentage of benzidine-positive cells was significantly higher than others concentration (Figure C). D. The expression of erythroid differentiation specific antigens on 8 µM Icaritin-treated K562 cells-flow cytometry assay results: upper panel: CD71 and control; bottom panel: glycophorin A (CD235) and control. E-a. Icaritin induced a prolonged activation of P-P38 in K562 cells until 8 days. P-JNK activation was also been observed, but diminished after 6 days. P-ERK protein was down-regulated in Icaritin-treated K562 cells (Western blotting results); E-b. Effects of Icaritin on MAPK pathways-time courses (0–24 hours). K562 cells were treated with 8 µM Icaritin in the indicated time interval (hours), total celluar extracts were prepared and subjected to Western blot assay to evaluate levels of phosphorylated forms of JNK, ERK and p38. β-actin was measured as control. The results showed Iaritin could up-regulate p-JNK level and inhibit p-ERK expression at a time-dependent manner, while p-p38 change was not significant. F. Effects of inhibition of MAPK pathways on Icaritin-induced K562 cells differentiation. (A). The K562 cells were treated with 8 µΜ Icaritin alone or together with 10 µΜ SB203580 from day 1 to day 6. Percentages of benzidine staining positive cells were evaluated on day 6. **p<0.01 vs control. (B). For p-p38 protein expression analysis, samples were harvested on day 6, and western blotting assay was performed. The result indicated that SB203580 could blunt Icaritin-induced P-38 phosphorylation.

Examination of Icaritin-treated K562 with light microscopy revealed that the survived K562 exhibited morphological changes such as reduction in cell volume indicating differentiation. Indeed, Icaritin-treated K562 exhibited higher hemoglobin level compared to untreated cells (Fig. 3A). The erythroid phenotype was also confirmed with Benzidine staining (Fig. 3B, 3C). We also analyzed the surface markers of erythroid with flow cytometry. The results showed that glycophorin A (CD235a) and transferrin receptor (CD71), erythroid specific antigens [22], [23], to a certain extent, had an increased expression in Icaritin treated K562 cells (Fig. 3D), indicating that Icaritin induces erythroid differentiation of K562.

The p38 has been shown to play a critical role in Icaritin-mediated cardiomyocyte differentiation in vitro [24]. Western blot results showed that Icaritin induced phosphorylation levels of p38 expression in K562 after treated for four days (Fig. 3E). The levels of phosphorylated JNK were also induced by Icaritin treatment (Fig. 3E), indicating that the p38 and JNK signal pathways were involved in Icaritin-induced K562 cell differentiation.

To determine the functions of the p38 and JNK in Icaritin-induced differentiation, K562 were treated with Icaritin alone or together with a p38 inhibitor, SB203580. The erythroid differentiation in these cells was assessed by benzidine staining and MAPK-related proteins were evaluated on day 6 with Western blotting. Figure 3F show that Icaritin-induced erythroid differentiation was abolished by SB203580 treatment, while SB203580 alone had no effect (Fig. 3F). Similarly, the increased expression of P-P38 following Icaritin treatment was inhibited in the presence of SB203580, while P-38 protein expression was not influenced by SB203580 (Fig. 3F).

### 4. Icaritin has no effect on Bcr/Abl expression

**Figure 4:**
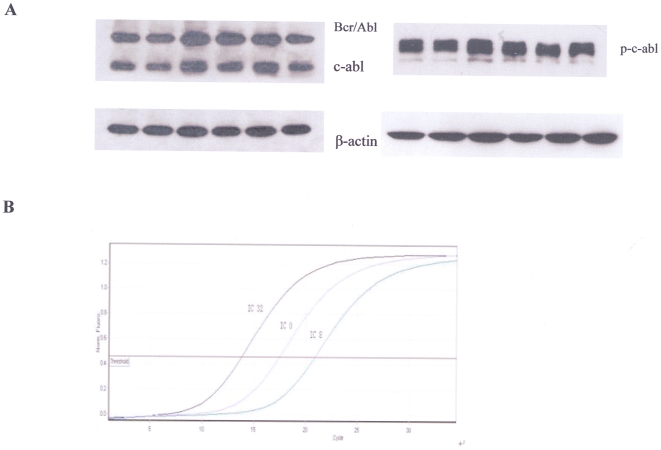
Effect of Icaritin on Bcr/Abl protein or mRNA expressions on K562 cells. A. K562 cells were incubated with different Icaritin concentrations (0 µM, 4 µM, 8 µM, 16 µM, 32 µM, 64 µM) for 45 h, and then lyzed and separated by SDS-PAGE. Proteins were blotted on PVDF membrane. c-Abl, p-c-abl and, Bcr/Abl protein levels were detected via immunoblot. β-actin was used as internal control. Results showed Icaritin had no influence on Bcr/Abl, c-Abl and phosphorylated c-Abl protein expression. B. Quantitative real-time RT-PCR analysis for Bcr/Abl mRNA level. The results indicated that compared with untreated K562 cells, Icaritin-treated K562 cells (both 8 µM and 32 µM) had no significant effect on the expression of Bcr/Abl mRNA.

Since Icaritin showed a similar effect on cell proliferation as Imatinib, we assessed its influence on Bcr/Abl fusion protein of K562. Our result showed that Icaritin at various concentrations had no influence both on c-Abl protein or phosphorylated c-Abl expression (by western blot) (Fig. 4A) and mRNA levels of Bcr/Abl (by Real-time PCR) (Fig. 4B), indicating that Icaritin anti-leukemic activity was not related to Bcr/Abl expression,and failed to demonstrate any significant alteration of Bcr/Abl phosphorylation level.

### 5. Icaritin inhibits MAPK/ERK/JNK downstream signaling and diminishes Jak2/Stat3/Akt expression

**Figure 5:**
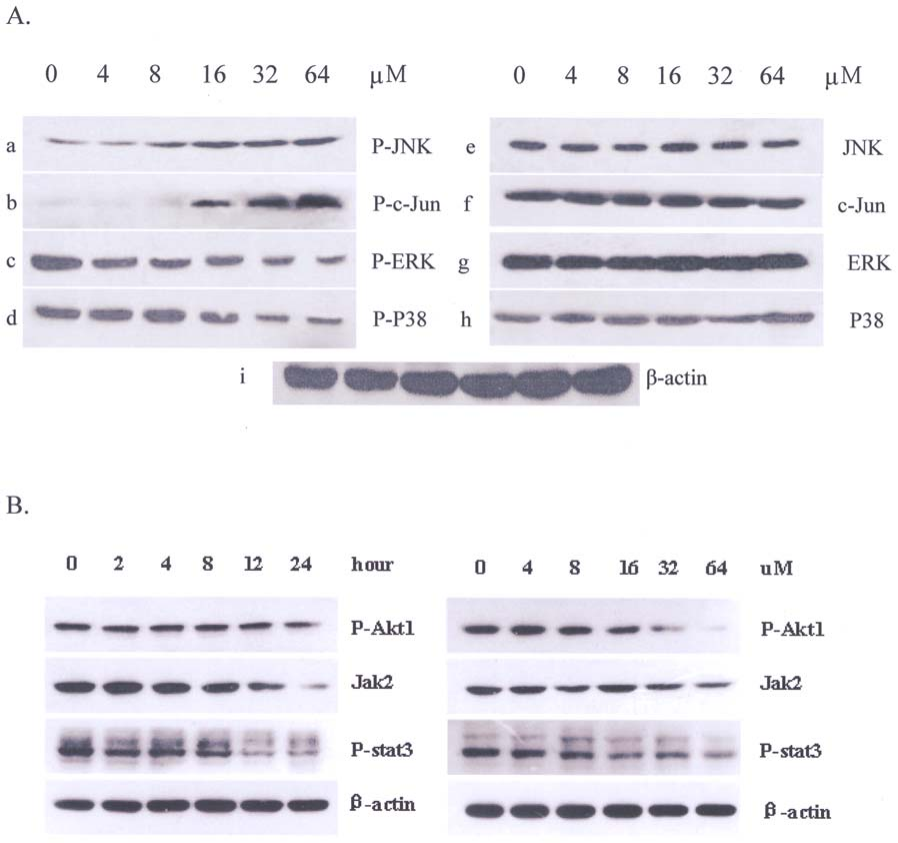
Effects of Icaritin on MAPK/ERK/JNK signaling pathways and Jak2/Stat3/Akt axes. A. Icaritin could up-regulate phospho-JNK, or phospho-C-Jun (Fig.5A- a, b.), and down-regulate phosphor-ERK, phospho-P-38 expressions (Fig.5A-c, d) with dose-dependent manner. While JNK, C-jun, ERK, p38 (Fig.5A-e, f, g, h) expressions were not influenced by Icaritin. Normalization was performed using β-actin (Fig.5A-i). B. Icaritin diminishes constitutive activation of JAK-2, p-Stat3 and p-Akt in K562 cells at time- or dose-dependent manner. K562 cells were treated with 8 µM Icaritin for 1-24 hours (left panel), or with different concentrations of Icaritin (0–64 µM) (right panel). After which the cells were lysed and subjected to Western blot analysis to monitor expression of JAK-2, phosphorylated Stat3 and Akt.

To further characterize the mechanisms involved in the proapoptotic action or proliferation- inhibiting of Icaritin, we analyzed its effect on the main signaling pathways related to proliferation or apoptosis regulation. After treated with Icaritin for 48 h, western blot was done. Our results demonstrated that Icaritin might up-regulate phospho-JNK and phospho-C-Jun expression (Fig. 5A: a, b); however, it abolished JNK or C-JUN basal activation (Fig. 5A: e, f). Interestingly, although Icaritin failed to affect ERK or P-38 expression (Fig. 5A: g, h), it could inhibit the activation of phospho-ERK or phospho-P38 (Fig. 5A: c, d). Thus, the main effect of Icaritin is to abolish P-ERK, P-P38 constitutive activation in K562.The time-dependent effect of Icaritin on above-mentioned proteins were also examined. As shown in Fig. 3E-a, Icaritin treatment resulted in a significant increase in phospho-JNK, and a down-regulated expression in phospho-ERK, which were consistent with the changes of dose-dependent effect of Icaritin.It has been reported that activation of Jak-2 is very important for Bcr/Abl-mediated oncogenicity and may be a potentially therapeutic target [25], [26]. Akt kinase is also constitutively active in the chronic phase of CML, blast crisis of CML, and K562 cell line[27] and activation of Stat3 has been implicated in survival signaling downstream of Bcr/Abl[28]. To assess the effects of Icaritin on Jak2/Stat/Akt signal network, Western blot analysis was completed. As shown in Fig. 5B, Icaritin both reduced Jak-2, p-Stat3 and p-Akt expression at a dose- and time-dependent manner, suggesting that Icaritin also disturbs Jak2/Stat3/Akt signal pathway and facilitates the growth-arrest of leukemia cells. These results, taken together, indicated that Icaritin induced CML cell apoptosis was associated with MAPK/ERK/JNK signaling regulation, and, at least in part, Jak2/Stat3/Akt signaling interference may play a significant role in inhibiting cell proliferation and survival of Bcr/Abl+ leukemia cells.

### 6. Icaritin inhibits growth of CML *in vivo*

**Figure 6:**
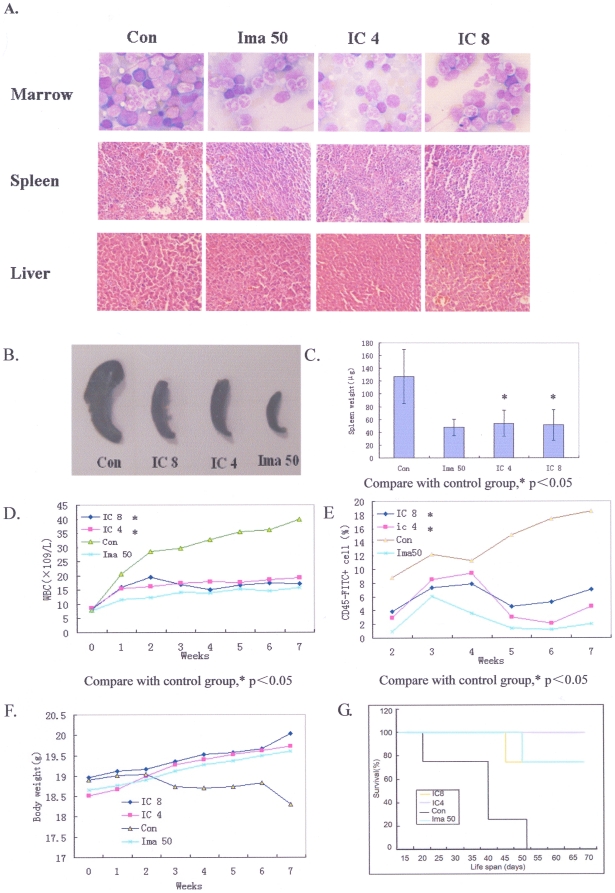
*In vivo* anti-leukemic effects of Icaritin. A. Icaritin reduces K562 leukemic cells infiltration in mice bone marrow (up panel, Wight-Gimesa, ×1000 staining). From left to right, the bone marrow smear is hypercellularity and granulocytic hyperplasia with increased basophils and eosinophils in untreated mouse (Con); while in Imatinib- or Icaritin-treated mouse, bone marrow show a decreased granulocytic number and the inhibition of hyperplasia (Ima50; IC4; IC8). Spleen (middle panel), as well as liver tissue section (bottom panel). Hematoxylin-Eosin (HE) staining (×200). Splenic and hepatic patholoic section show an increased infiltration of leukemia cells in hepatic sinus and periportal or splenic sinus with sinusoidal extension in untreated-mouse (Con), the infiltraion of leukemic cells is more evident in spleen than liver. In Imatinib- or Icaritin-treated mouse, the infiltration of leukemic cells was decreased and the change of tissue structure is not significant (Ima50; IC4; IC8). B. C. In Icaritin-treated mice, the spleen weight and size decreased significantly both 4 mg/kg Icaritin dose and 8 /kg Icaritin dose. D. Changes of white blood cells (WBC) count in peripheral blood for Icaritin-treated mice and controls. E. Changes of human WBC antigen (CD45) levels in Icaritin-treated mice and controls. F. Changes of body weight of mice treated with Icaritin or imatinib. G. Icaritin prolongs lifespan of mice bearing K562 cells.

To evaluate the anti-leukemia effect of Icaritin *in vivo*, NOD-SCID mice receiving 2×10^6^ K562 cells inoculation were treated with Icaritin. Mice treated with vehicle alone, splenomegaly was very obvious and histological examination revealed extensive infiltrations of leukemia cells in spleen, bone marrow and liver (Fig. 6A), indicating K562 cells were able to invade these tissues. Icaritin dramatically reduced these disseminated infiltrations as effective as Imatinib (Fig. 6A). In Icaritin- and Imatinib-treated mice, the weight and size of spleens were evidently less than those of the group treated with vehicle (P<0.05)(Fig. 6 B.C). Both directly counting and CD45 antigen assay showed that the numbers of WBC in peripheral blood in vehicle treated mice were significantly higher than those in Icaritin-treated mice (P<0.05) (Fig. 6D.E). However, Icaritin caused neither bone marrow suppression nor weight loss (Fig. 6F); indicating Icaritin has no general cytotoxic effects.

We also found that Icaritin could prolong median survival time of leukemia-bearing mice (39.5±14.4 days for vehicle treated mice versus 61.75±10.5 days and 67±0 days for mice treated with Icaritin at 8 mg/kg/day and 4 mg/kg/day, respectively), that was similar to the effect of Imatinib (Fig. 6G).

## Discussion

In present study, we found Icaritin, a compound purified from traditional herb medicine exhibited a potent anti-leukemia activities towards established CML cell line-K562 and primary bone marrow cells (including CD34+ cells) from CML patients. Icaritin effectively inhibited K562 growth *in vitro*. At concentration of 8 µM, Icaritin could lead to more than 50% of growth inhibition of K562. More importantly, we also observed that Icaritin exhibited strong efficacies on primary bone marrow cells from CML-CP and CML-BC patients while had no effect on growth and proliferation of normal bone marrow cells (Fig. 1B-b), indicating Icaritin has low or no general cytotoxic effect on normal hematopoiesis. Consistent with this, Icaritin showed potent effects with low adverse reactions such as weight loss *in vivo*. We also checked the effects of Icaritin on Imatinib-resistant cells line and Imatinib-resistant primary cells from one CML patient, our results suggested that Icaritin evidently inhibited the growth of both Imatinib-resistant cells line and Imatinib-resistant primary cells. Furthermore, we also confirmed that Icaritin could induce Imatinib-resistant cells apoptosis. Although the results are preliminary; this will be a new clue for searching an alternative agent in overcoming Imatinib-resistance of Bcr/Abl+ cells.

Accumulating evidence indicated that many types of cancer, including leukemia, originate from and are maintained by a small of cancer stem/progenitor cells. These cancer stem/progenitor cells are often resistant to most therapeutic strategies. In this study, we enriched CML stem/progenitor cells from 3 patients with CML-BC using CD34 selection kit and successfully isolated CD34+ leukemia cells (the yield: 89.37%±6.79%). Here, we showed that Icaritin could effectively inhibit leukemic stem/progenitor cells proliferation and induce apoptosis, and thus suggesting that the effect of Icarintin on anti-leukemia activity may preferentially target to leukemic stem/progenitor cells.

It has been characterized that both extrinsic and intrinsic apoptotic pathways are involved in the activation of effectors caspases (casp-3, casp-2 and casp-7). The extrinsic pathways is initiated by binding death receptors, such as CD95/Fas, TNF or TRAIL receptor to activate caspase-8 and caspese-10, which in turn cleave and activate effectors caspases[29], [30]. The involvement of intrinsic apoptotic pathway is more often events especially in cancer cells, which is characterized by the disruption of the mitochondrial membrane and proteins release [31]. Our results demonstrated that Icaritin was able to induce apoptosis, both in K562 and primary CML cells. Icaritin inhibited Bcl-2 expression and up-regulated Bax expression, which resulted in a lower ratio of Bcl-2/Bax. We also found that cytochrome C levels were up-regulated, caspase-9 and caspase-3 were cleavaged and activated, Apaf-1 expression was down-regulated following Icaritin treatment, indicating the mitochondrial permeability is changed. Based on the observations, we suggested that mitochondrial-mediated caspase cascade pathway plays a major role in Icaritin-induced apoptosis.

K562 have been widely used as a model for leukemia differentiation. It is known to be induced to differentiate along either erythroid or megakaryocytic lineage [20]. In our experiments, both morphologic and phenotypic analysis revealed that after Icaritin treatment for 6 to 8 days, a significant number of K562 exhibited erythroid-like features, including the change of cell volume, increased Hb concentration, RBC benziding staining and expression of erythroid specific markers, such as glycophorin-A (CD235a) and transferring receptor (CD71). It has been shown erythroid differentiation is a tightly regulated process that requires specific transcription factors [32], [33]. Accordingly, it has been reported that hydroxyurea induced erythroid differentiation of K562, which was associated with the activation of the p38 MAPK pathway [34]. Davidson and Morange showed activation of the p38 pathway was necessary for cardiomyogenesis of the P19 embryonic carcinoma cell line during early stages [35]. Recently, Ding et al showed that Icaritin-induced cardiomyocyte differentiation of murine embryonic stem cells was associated with enhanced phosphorylation of p38[24]. Consistent with these observations, we found that Icaritin significantly induced phosphorylation levels of p38 during K562 cell differentiation toward erythroid lineage, which was blocked by p38 inhibitor SB203580. We concluded Icaritin was able to induce CML cell differentiation presumably through the p38 pathway.

The potent anti-leukemia efficacy of Icaritin in murine model demonstrated that Icaritin was able to reduce the infiltration of leukemia cells and alleviate the load of leukemia cells in peripheral blood and spleen; effects were similar to those of Imatinib. Survival study also showed that Icaritin was able to significantly prolong the lifespan of mice loaded with leukemia. We found that Icaritin failed to influence Bcr/Abl expression in K562. However, whether Icaritin has influence on the downstream signal pathways of Bcr/Abl need to be further proven.

The anti-apoptotic activity of Bcr/Abl contributes greatly to the development of CML. Bcr/Abl may function either by enhancing the proliferation potential of hematopoietic progenitors or by protecting these progenitor cells from apoptosis [36], [37]. Accumulating evidence shows that the constitutive tyrosine kinase activity of Bcr-Abl is essential for its leukemogenic activity [38]. Bcr-Abl-mediated signal transduction pathways could interfere with various cellular physiological processes, including cells proliferation, adhesion, and apoptosis [39], [40]. These processes are thought to involve in intracellular signaling pathways, such as MEK/ERK, JNK/SAPK and p38MAPK [41], [42], [43]. It has been shown that the STAT family of transcription factors plays important role in transformation and antiapoptotic signaling stemming from constitutive activation of Bcr/Abl kinase [44], [45]. As a driving force for CML, the activated tyrosine kinase of Bcr/Abl is able to stimulate the Janus kinase (Jak) 2 pathway [25]. Furthermore, PI3K/Akt pathway has emerged as one of the essential signaling mechanisms in Abl leukemogenesis as its downstream effectors are responsible for propagating the signals to promote myeloid and lymphoid transformation[46]. To assess whether the effect of Icaritin on anti-leukemia is involves in the inactivation of Jak-2/Stat-3/Akt axes, western blot was used for evaluating the expression of Jak-2, p-Stat-3 and p-Akt protein. It is noteworthy that the exposure of K562 cells to Icaritin resulted in diminished phospho-p38 or phospho-ERK expression, and induced activation of phospho-JNK or phospho-c-Jun, which contributes apparently to the apoptosis of Bcr/Abl+ cells. Significantly, Icaritin can obviously down-regulate the expression of Jak-2, phospho-Stat3 and phospho-Akt protein at a dose- and time-dependent manner, suggesting that the interruption of Jak-2/Stat-3/Akt signal network by Icaritin contributes to the growth-inhibition and diminished survival of K562 cells. Collectively, these observations suggest that transcriptional repression by Icaritin lowers the proliferation potential for Icaritin-mediated growth-inhibition of Bcr/Abl+ leukemia cells. On the other hand, the possibility that the action of Icaritin as an apoptosis-activator contribute to a diminished apoptotic threshold cannot be completely excluded. However, exact molecular mechanisms on anti-leukemia activity of Icaritin still need to be elucidated.

In conclusion, we have documented for the first time the anti-CML effects of Icaritin *in vitro* and *in vivo*. Our findings have shown that Icaritin was able to inhibit CML cell growth, and induce apoptosis and differentiation. The underlying mechanisms of Icaritin anti-CML activity are involved in the inhibition of MAPK/ERK/JNK signals and down-regulated kinase activity of Jak-2/Stat-3/Akt signal network. Further investigation of this novel anti-CML agent may offer insights into the pathogenetic mechanisms of CML and provide a new approach for CML treatment and a reversal to Imatinib-resistance.

## References

[1] Buchdunger E, Zimmermann J, Mett H, Meyer T, Muller M (1996). Inhibition of the Abl protein-tyrosine kinase in vitro and in vivo by a 2-phenylaminopyrimidine derivative.. Cancer.

[2] O'Brien SG, Guilhot F, Larson RA, Gathmann, Sc.I M, Baccarani M (2003). Imatinib compared with interferon and low-dose cytarabine for newly diagnosed chronic-phase chronic myeloid leukemia.. N Engl J.

[3] Hughes TP, Kaeda J, Branford S, Rudzki Z, Hochhaus A (2003). Frequency of major molecular responses to imatinib or interferon alfa plus cytarabine in newly diagnosed chronic myeloid leukemia.. N Engl J Med.

[4] Talpaz M, Silver RT, Druker BJ, Goldman JM, Gambacorti-Passerini C (2002). Imatinib induces durable hematologic and cytogenetic responses in patients with accelerated phase chronic myeloid leukemia: results of a phase 2 study.. Blood.

[5] Sawyers CL, Hochhaus A, Feldman E, Goldman JM, Miller CB (2002). Imatinib induces hematologic and cytogenetic responses in patients with chronic myelogenous leukemia in myeloid blast crisis: results of a phase II study.. Blood.

[6] Druker BJ, Guilhot F, O'Brien SG, Gathmann MScI, Kantarjian H (2006). Five-year follow-up of patients receiving imatinib for chronic myeloid leukemia.. N Engl J Med.

[7] Calabretta B, Perrotti D (2004). The biology of CML blast crisis.. Blood.

[8] Shet AS, Jahagirdar BN, Verfaillie CM (2002). Chronic myelogenous leukemia: mechanisms underlying disease progression.. Leukemia.

[9] Weisberg E, Manley PW, Breitenstein W, Brüggen J, Cowan-Jacob SW (2005). Characterization of AMN107, a selective inhibitor of native and mutant Bcr-Abl.. Cancer Cell.

[10] Wang ZQ, Lou YJ (2004). Proliferation-stimulating effects of icaritin and desmethylicaritin in MCF-7 cells.. Eur J Pharmacol.

[11] Liu H, Ji H, Zhang CY (2006). Protective effect of Icariin on primary cultured neonatal rat cardiomyocytes treated with isoproterenol-induced injury.. Chin pharmacol Bull(12).

[12] He W, Sun H, Yang B, Zhang D, Kabelitz D (1995). Immunoregulatory effects of the herba Epimediia glycoside icariin.. Arzneimittelforschung.

[13] Zhang ZH, Xu H, Liu MB (2006). Experimental study on Icariin induced apoptosis of cultured liver cancer cell smmc-7721.. Practical clinical medicine.

[14] Zhang JW, Zhou YF, Wen XM (2006). Icariin reverses malignant phenotype of gastric carcinoma cells.. Chin Experiment Surgical Journal.

[15] Yi Z, Cui Z, Zhang L (1997). Effects of icariin on the differentiation of HL-60 cells.. Zhong hua zhong liu za zhi.

[16] Li GX, Zhang L, Wang Y (2002). Effects of icariin on apoptosis inducement and oncogene expression for HL-60 cells.. Chin J Hematol.

[17] Ferrao PT, Frost MJ, Siah SP, Ashman LK (2003). Overexpression of P-glycoprotein in K562 cells does not confer resistance to the growth inhibitory effects of imatinib (STI571) in vitro.. Blood.

[18] Zhang GS Tu CQ, Zhang GY, Zhou GB, Zheng WL (2000). Indomethacin induces apoptosis and inhibits proliferation in chronic myeloid leukemia cells.. Leuk Res.

[19] Peng HL, Zhang GS, Liu JH, Gong FJ, Li RJ (2008). Dup-697, a specific COX-2 inhibitor, suppresses growth and induces apoptosis on K562 leukemia cells by cell-cycle arrest and caspase-8 activation.. Ann Hematol.

[20] Belhacène N, Maulon L, Guérin S, Ricci JE, Mari B (1998). Differential expression of the Kell blood group and CD10 antigens: two related membrane metallopeptidases during differentiation of K562 cells by phorbol ester and hemin.. FASEB J.

[21] Mensink E, Van de locht A, Schattenberg A, Linders E, Shaap N (1998). Quantitation of minimal residual disease in Philadelphia chromosome positive chronic myeloid leukemia patients using real-time quantitative RT-PCR.. Br J Haematol.

[22] Andersson LC, Gahmberg CG, Teerenhovi L, Karhi KK, Gahmberg CG (1979). Glycophorin A as a cell surface marker of early erythroid differentiation in acute leukemia.. Int J Cancer.

[23] Uddin S, Ah-Kang J, Ulaszek J, Mahmud D, Wickrema A (2004). Differentiation stage-specific activation of p38 mitogen-activated protein kinase isoforms in primary human erythroid cells.. Proc Natl Acad Sci USA.

[24] Ding L, Liang XG, Hu Y, Zhu DY, Lou YJ (2008). Involvement of p38MAPK and reactive oxygen species in icariin-induced cardiomyocyte differentiation of murine embryonic stem cells in vitro.. Stem Cells Dev.

[25] Xie S, Wang Y, Liu J, Sun T, Wilson MB (2001). Involvement of Jak-2 tyrosine phosphorylation in Bcr-Abl transformation.. Oncogene.

[26] Samanta AK, Lin H, Sun T, Kantarjian H, Arlinghaus RB (2006). Janus Kinase 2: a critical target in chronic myelogenous leukemia.. Cancer Res.

[27] Kawauchi K, Ogasawara T, Yasuyama M, Ohkawa S (2003). Involvement of Akt kinase in the action of STI571 on chronic myelogenous leukemia cells.. Blood Cells Mol Dis.

[28] Coppo P, Dusanter-Fourt I, Millot G, Nogueira MM, Dugray A (2003). Constitutive and specific activation of STAT3 by BCR/ABL in embryonic stem cells.. Oncogene.

[29] Cohen GM (1997). Caspases: the executioners of apoptosis.. Biochem J.

[30] Nicholson DW (1999). Caspase structure, proteolytic substrates, and function during apoptotic cell death.. Cell Death Differ.

[31] Johnstone RW, Ruefli AA, Lowe SW (2002). Apoptosis: a link between cancer genetics and chemotherapy.. Cell.

[32] Bartel FO, Higuchi T, Spyropoulos DD (2000). Mouse models in the study of the Ets family of transcription factors.. Oncogene.

[33] Zhu J, Emerson SG (2002). Hematopoietic cytokines, transcription factors and lineage commitment.. Oncogene.

[34] Park JI, Choi HS, Jeong JS, JY, Kim IH (2001). Involvement of p38 kinase in hydroxyurea-induced differentiation of K562 cells.. Cell Growth Differ.

[35] Davidson SM, Morange M (2000). Hsp25 and the p38 MAPK pathway are involved in differentiation of cardiomyocytes.. Dev Biol.

[36] McGahon A, Bissonnette R, Schmitt M, Cotter K, Green D (1994). BCR-ABL maintains resistance of chronic myelogenous leukemia cells to apoptotic cell death.. Blood.

[37] Advani AS, Pendergast AM (2002). Bcr-Abl variants: biological and clinical aspects.. Leuk Res.

[38] Lugo TG, Pendergast AM, Muller AJ, Witte O (1990). Tyrosine kinase activity and transformation potency of bcr-abl oncogene products.. Science.

[39] Deininger MW, Goldman JM, Melo JV (2000). The molecular biology of chronic myeloid leukemia.. Blood.

[40] Shet AS, Jahagirdar BN, Verfaillie CM (2002). Chronic myelogenous leukemia: mechanisms underlying disease progression.. Leukemia.

[41] Puil L, Liu J, Gish G, Mbamalu G, Bowtell D (1994). Bcr-Abl oncoproteins bind directly to activators of the Ras signalling pathway.. EMBO J.

[42] Burgess GS, Williamson EA, Cripe LD, Litz-Jackson S, Bhatt JA (1998). Regulation of the c-jun gene in p210 BCR-ABL transformed cells corresponds with activity of JNK, the c-jun N-terminal kinase.. Blood.

[43] Yu C, Krystal G, Varticovksi L, McKinstry R, Rahmani M (2002). Pharmacologic mitogen-activated protein/extracellular signal-regulated kinase kinase/mitogen-activated protein kinase inhibitors interact synergistically with STI571 to induce apoptosis in Bcr/Abl-expressing human leukemia cells.. Cancer Res.

[44] Buettner R, Mora LB, Jove R (2002). Activated STAT signaling in human tumors provides novel molecular targets for therapeutic invention.. Clin Cancer Res.

[45] Spiekermann K, Pau M, Schwab R, Schmieja K, Franzrahe S (2002). Constitutive activation of STAT3 and STAT5 is induced by leukemic fusion proteins with protein tyrosine kinase activity and is sufficient for transformation of hematopoietic precursor cells.. Exp Hematol.

[46] Skorski T, Bellacosa A, Nieborowska-Skorska M, Majewski M, Martinez R (1997). The transformation of hematorpoietic cells by Bcr/Abl, requires activation of a PI-3k/Akt-dependent pathway.. EMBO J.

